# Development and application of a novel scintillation gel‐based 3D dosimetry system for radiotherapy

**DOI:** 10.1002/acm2.14615

**Published:** 2024-12-20

**Authors:** Hua Li, Haijing Jin, Liang He, Xuewen Yan, Hui Zhang, Deyuan Li

**Affiliations:** ^1^ Frontier Technology Center China Institute for Radiation Protection Taiyuan Shanxi China; ^2^ Shanxi Provincial Key Laboratory for Translational Nuclear Medicine and Precision Protection Taiyuan Shanxi China

**Keywords:** 3D dosimetry, quality assurance, radiotherapy, scintillation gel

## Abstract

**Purpose:**

This study introduced a novel 3D dosimetry system for radiotherapy in order to address the limitations of traditional quality assurance methods in precision radiotherapy techniques.

**Methods:**

The research required the use of scintillation material, optical measurements, and a dose reconstruction algorithm. The scintillation material, which mimics human soft tissue characteristics, served as a both physical phantom and a radiation detector. The dose distribution inside the scintillator can be converted to light distributions, which were measured by optical cameras from different angles and manifested as pixel values. The proposed dose reconstruction algorithm, LASSO‐TV, effectively reconstructed the dose distribution from pixel values, overcoming challenges such as limited projection directions and large‐scale matrices.

**Results:**

Various clinical plans were tested and validated, including a modified segment from the SBRT plan and IMRT clinical plan. The dosimetry system can execute full 3D dose determinations as a function of time with a spatial resolution of 1–2 mm, enabling high‐resolution measurements for dynamic dose distribution. Comparative analysis with mainstream device MapCHECK2 confirmed the accuracy of the system, with a relative measurement error of within 5%.

**Conclusions:**

Testing and validation results demonstrated the dosimetry system's promising potential for dynamic treatment quality assurance.

## 1 INTRODUCTION

Radiotherapy is currently one of the most widely used methods for cancer treatment.^1^, ^2^, ^3^ Using China as an example, statistics show that in 2020, China had approximately 1,538 radiotherapy units with 32,978 practitioners and over 2,600 radiation therapy devices and treated more than 2 million patients annually.^4^ Radiotherapy technology has evolved from traditional Co‐60 treatment machines to accelerators, expanding radiation types from electrons and photons to protons and heavy ions. Continuing this evolution, dynamic and multi‐angle irradiation techniques have been developed and applied, enabling precision at sub‐millimeter levels. Moreover, precision radiotherapy techniques, including intensity‐modulated radiation therapy (IMRT), volumetric modulated arc therapy (VMAT), and tomotherapy, have emerged.^5^, ^6^, ^7^, ^8^

To ensure the accurate implementation of treatment plans, complex quality assurance methods are a prerequisite for precision radiotherapy techniques. Presently, traditional radiotherapy quality assurance is primarily based on point or 2D dose verification, typically using dose measurement methods such as ionization chambers and semiconductor detectors, which are considered low‐sampling, as well as films, which can only measure planar dose.^9^ Nevertheless, these methods only provide low‐resolution point or planar dose data, which cannot meet the increasing demand for 3D dose verification in complex radiotherapy techniques. The medical physics community recognizes that only high‐resolution 3D dosimeters can provide comprehensive validation of dynamic dose delivery.

In the field of 3D dose verification, various measurement technologies are available, including polymer gel dosimetry,^10^, ^11^ radiochromic dosimetry,^12^, ^13^ detector matrices,^14^, ^15^ scintillation dosimetry,^16^, ^17^, ^18^, ^19^ and so forth. These methods offer accurate and precise results, with spatial resolutions ranging from 0.5 to 5 mm. However, these technologies still have certain limitations regarding quality assurance. For example, polymer gel dosimetry requires additional complex and time‐consuming readout processes and can only provide cumulative dose information and thus lacks time‐resolved dose detection capabilities.^20^ Radiochromic dosimetry suffers from instability, as Fe^2+^ can diffuse after oxidation, reducing spatial resolution, and they are susceptible to temperature, light, and oxygen pressure. With detector matrices, the spatial resolution is limited by the spacing between detectors, and dose between intervals is obtained through interpolation, introducing significant errors in regions with large dose gradients.

In recent years, many research groups have been investigating the use of plastic or liquid scintillators for 3D dose measurement.^21^, ^22^, ^23^, ^24^ While this technology has shown promise by providing accurate dose measurements throughout the entire 3D volume, its practical application is limited by factors such as the necessity for component rotation during data acquisition.^25^, ^26^, ^27^, ^28^

**FIGURE 1:**
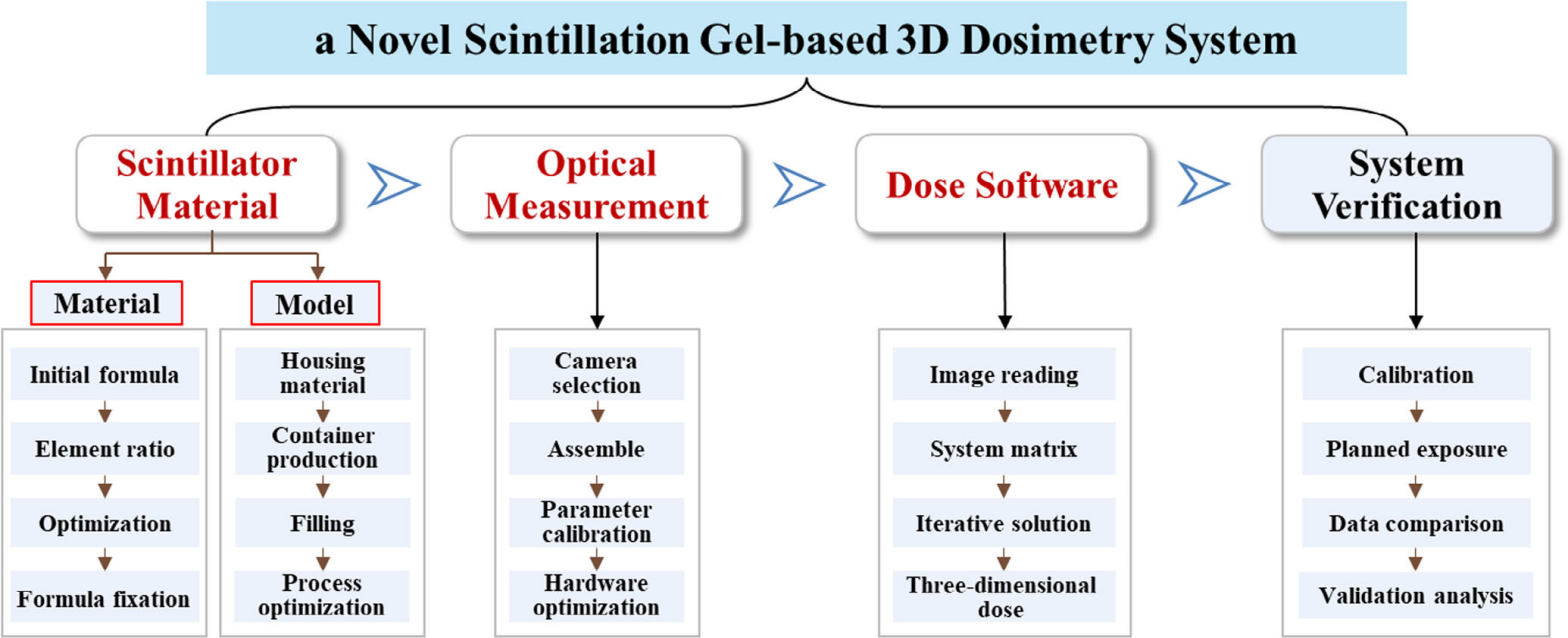
Research framework of this study, including various steps in the creation, optimization and evaluation of scintillation gel‐based 3D dosimetry system.

In the present study, we examined the feasibility of a time‐resolved 3D dosimetry system for radiotherapy QA through three components: scintillation material,^29^ optical measurements, and dose reconstruction,^30^ as illustrated in Figure 1. The primary advantage is that the independently developed scintillation gel material mimics human soft tissue characteristics. This material can be utilized for both physical phantoms and radiation detection. Furthermore, we proposed a 3D dose reconstruction algorithm, LASSO‐TV,^30^ based on the least‐angle regression method and total variation (TV) constraint. This algorithm effectively addresses challenges such as limited projection directions and large‐scale matrices. The 3D dosimetry system enables accurate measurements of soft tissue equivalence, millimeter‐level high spatial resolution, time‐resolved measurements, and full 3D dose determination. Furthermore, the system was tested and validated on a modified segment from the stereotactic body radiotherapy (SBRT) plan and IMRT clinical plan.

## 2 METHODS AND MATERIALS

### 2.1 Scintillation gel material and physical phantom

In this study, organic emulsion gel materials containing benzene ring π electrons based on emulsion polymerization were developed. The preparation of the initial scintillation gel was achieved by incorporating a scintillator (2,5‐Diphenyloxazole, PPO) and a wavelength shifter (1,4‐Bis(2‐methylstyryl)benzene, Bis‐MSB).^29^ Based on the primary solvent used, prepared scintillation gel materials were classified into two categories: linear alkylbenzenes (LAB) and diisononyl naphthalene (DIN). The initial elemental compositions of these two types of gel materials are measured by elemental analyzer and provided in Table 1.

**TABLE 1:** Composition of the two types of gel materials.

	Fraction of mass/%		
Type	C	H	O
LAB Gel	52.5	10.7	36.8
DIN Gel	51.7	10.9	37.4

**FIGURE 2:**
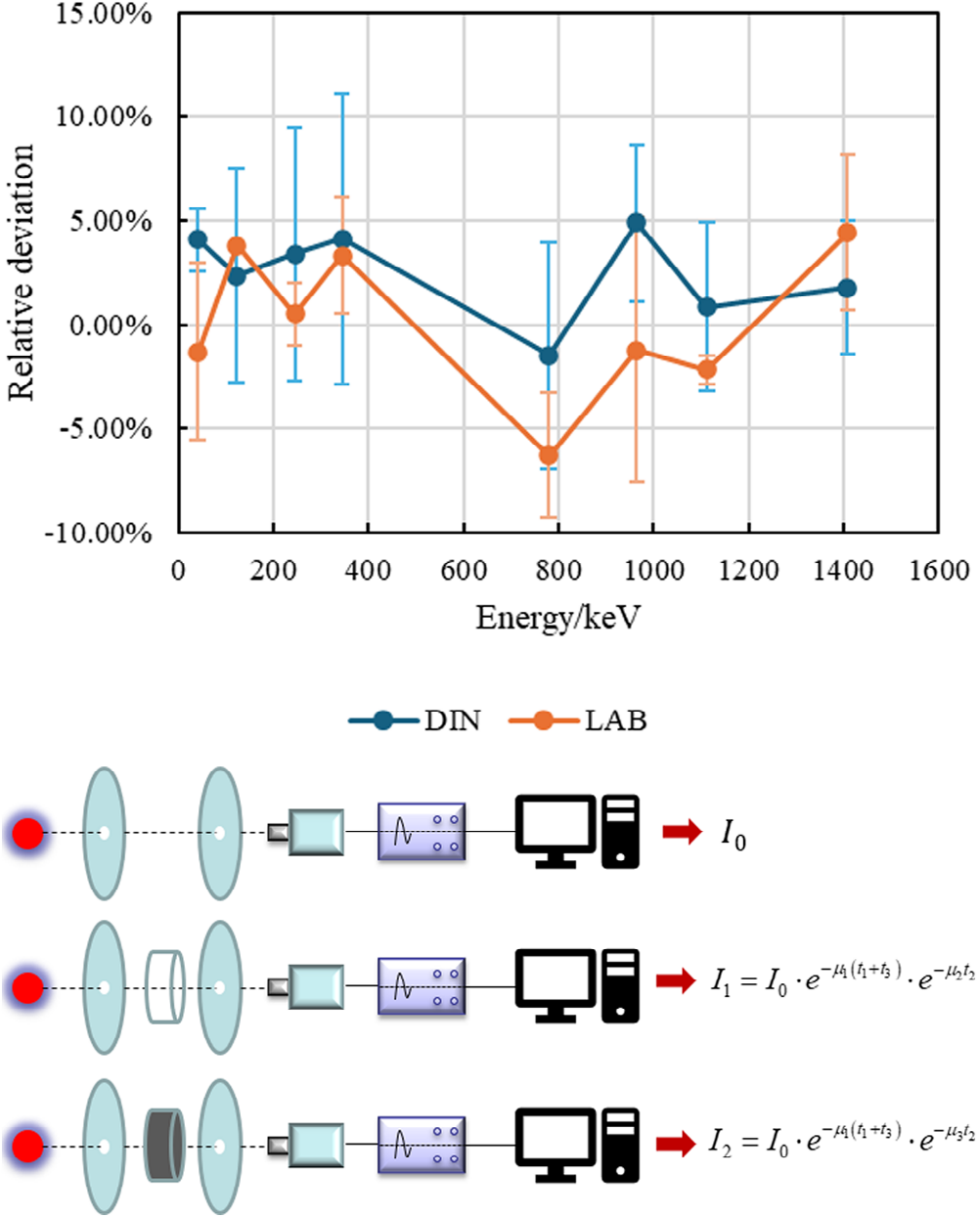
Relative deviation between the experimental results and ICRU‐recommended γ mass attenuation coefficient of the LAB and DIN gel, including the experimental procedure using Eu‐152 standard source and high‐purity germanium (HPGe) detectors. LAB, linear alkylbenzenes; DIN, diisononyl naphthalene.

Building upon the initial gel materials, this study focused on optimizing their tissue equivalence and scintillation luminescence properties. Testing of the initial scintillation gel showed significant deviations from the ICRU‐recommended human soft tissue parameters^31^ in the low‐energy range. To enhance the performance of the scintillation gel, a combined approach using Monte Carlo simulation and experimental testing was employed. An optimized formulation was proposed by adding 1% inorganic salt ZnCl_2_. The optimized scintillation gel showed deviations of less than 5% from the ICRU‐recommended γ mass attenuation coefficient in an energy range of 0.03–20 MeV, indicating excellent soft tissue equivalence, as shown in Figure 2. Through the method of controlling variables, the amounts of the scintillator PPO and the wavelength shifter Bis‐MSB in the gel were adjusted multiple times. Ultimately, after adjusting the content of the scintillator PPO to 0.33% and the wavelength shifter Bis‐MSB to 0.0056%, the scintillation gel's light yield reached its maximum, approximately 80% of that of the standard liquid scintillator. During testing light yield, measurements were taken at 3–4 different positions in each of three orthogonal planes. The results demonstrated consistent light output, with an average yield of 78.6% relative to the standard liquid scintillator.

**FIGURE 3:**
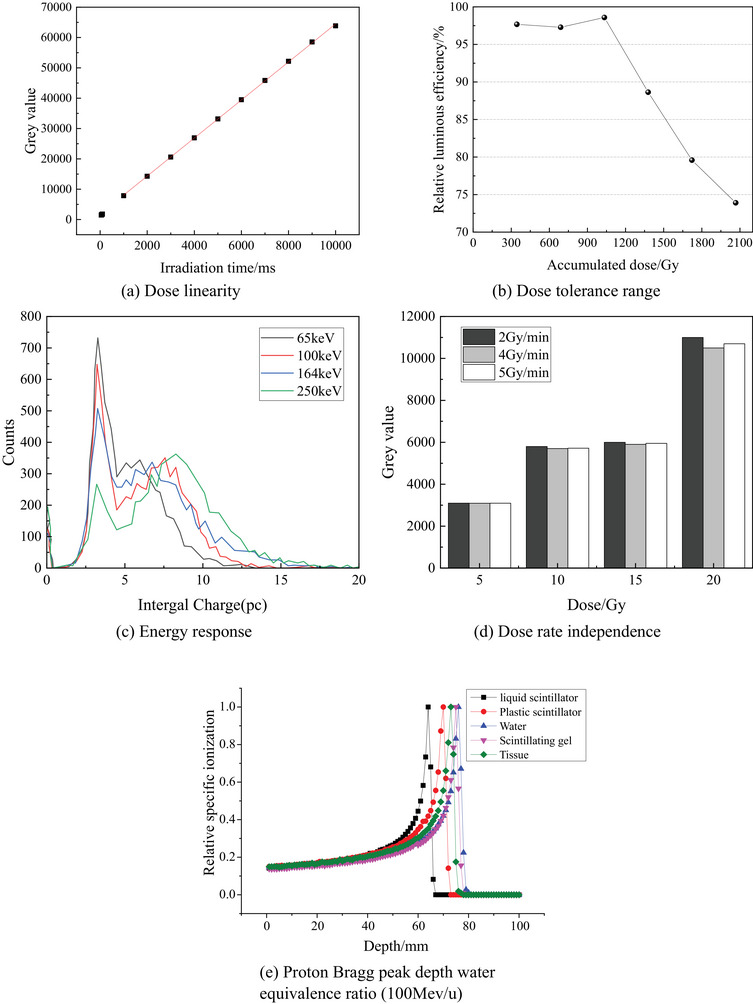
Performance test results of the scintillation gel for dosimetry.

Based on this optimization work, a final formulation for the scintillation gel was derived. Experimental testing confirmed its satisfactory dose linearity, maximum dose tolerance range, energy response, dose rate independence and proton Bragg peak depth water equivalence ratio, as shown in Figure 3.

**FIGURE 4:**
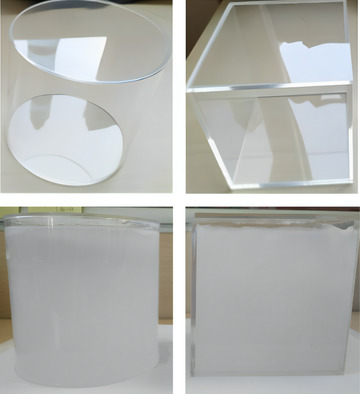
Cylindrical and cubic phantom shells without and with scintillation gel.

For the physical phantom, based on the preparation of the scintillation gel, transparency and chemical resistance tests were conducted, and polymethyl methacrylate (PMMA) material was finally selected for the phantom shell. The shell was manufactured in both cylindrical and cubic shapes, as shown in Figure 4. Based on the results of experimentation with parameters such as drying and dehumidification pretreatment, melting temperature, filling speed, cooling speed, cooling time, environmental temperature, humidity, and vacuum control, a technique for filling and molding the scintillation gel was successfully developed. This technique ensured the absence of air bubbles during the filling process.

### 2.2 Principle of dose reconstruction

The luminescence mechanism of scintillation gel^30^ is related to the electronic energy level structure of organic molecules: when incident particles excite electrons at the valence band energy level of the molecule, valence electrons transition to higher energy levels. After a brief period, the electrons de‐excite to different energy levels, simultaneously emitting fluorescence. This fluorescence can be collected and measured using optical cameras. In this study, three cameras distributed orthogonally were used to image the fluorescence inside the scintillator. The 3D dose distribution within the scintillator was divided into multiple dose voxels, with the dose data of each voxel having a correlation with the light intensity generated in that region. The light intensity was recorded by the camera sensors and manifested as pixel values. For each camera, the mathematical expression for the dose reconstruction equation was^28^:

(1) p=A·D,

where $p\;=\{p_{j}:j\in \left\{1,\text{…},n\right\}\}$ represents the vector of pixel values, n represents the number of the employed camera sensor pixels, $D\;=\{D_{i}:i\in \;\left\{1,\text{…},m\right\}\}$ represents the vector of the 3D dose distribution, m represents the number of dose voxels within the scintillator volume, and A represents the system response matrix of dimension n×m, where each element $A_{ji}$ represents the contribution of the i‐th dose voxel to the j‐th pixel.

The weighted dose integral *p_j_* for each camera sensor pixel was calculated from its collected pixel intensity *I_j_*. The calculation formula was as follows:

(2) $$p_{j}=\frac{I_{j}}{I_{j,ref}}\;\cdot A_{j,*}\cdot {\vec{D}}_{ref},$$

where “ref” refers to the standard reference radiation field with a known 3D dose distribution, ${\vec{D}}_{ref}$ represents the 3D dose distribution vector of the standard reference radiation field. $A_{j,*}$ represents the j‐th row of the projection matrix A, and $I_{j,ref}$ represents the intensity collected by the sensor pixel corresponding to the standard reference radiation field. In the standard reference radiation field, ${\vec{D}}_{ref}$ is known and has high precision. Nevertheless, its error may introduce bias into the calculation of the weighted dose integral *p_j_*, thus affecting the calculation of the 3D dose distribution.

Furthermore, the influence of optical scattering needs to be considered. Optical scattering is the result of multiple absorption and re‐emission processes within the scintillator volume when light is emitted from a point source throughout the entire volume of the scintillator. This behavior was simulated as a uniform background of scattered scintillation light in this study. The background was proportional to the total dose deposited within the scintillator volume.

### 2.3 Calculation of the system response matrix

The accuracy of the 3D dose reconstruction system is determined by the system response matrix A. In this study, the optical software ZEMAX^32^ was employed to simulate and calculate the system response matrix. ZEMAX employed nonsequential mode for ray tracing, where a ray does not have a predefined path. When emitted and projected onto any object in the optical path, rays can undergo various physical processes, such as reflection, refraction, diffraction, scattering, and splitting into sub‐rays. This mode, which closely resembles real‐world scenarios, can be used to simulate the propagation and imaging of light within the scintillator.

ZEMAX OpticStudio15 was used to model the camera system, and simulations were conducted for the MVL‐KF3528M‐12MPE lens used in the 3D scintillation measurement platform. This lens has a focal length of 35 mm and a lens distortion of 0.02%. After specifying geometric parameters and determining the light source plane and imaging plane, imaging analysis was employed to simulate imaging. To enable batch ray tracing, the ZEMAX programming language was used to make modifications to the light source plane parameters. A total of 10^6^ rays were simulated to propagate from different object planes to the image plane, yielding the response of different pixels to dose voxels (i.e., the system response matrix A).

### 2.4 Iterative solution of the dose reconstruction equation

The process of dose reconstruction involves solving an overdetermined system of equations. In general, overdetermined systems have no exact solution and require finding an approximate solution. For the practical problem of dose reconstruction using three camera angles, the system response matrix is sparse. This study proposed the LASSO‐TV model for solving the dose reconstruction equation.^30^ LASSO stands for least absolute shrinkage and selection operator.^19^ Its basic approach is to minimize the sum of squared residuals under the constraint that the sum of the absolute values of the regression coefficients is less than a constant. This causes some regression coefficients to strictly equal zero, leading to a highly interpretable model suitable for sparse reconstruction scenarios. Moreover, to suppress noise during the iterative process, and considering that the dose distribution is continuous, smooth, and nonnegative, the reconstruction result can undergo TV minimization with nonnegativity constraints. The solution model for this method is expressed as:

(3) $$D^{\#}=\arg\min_{D\geq 0}\;\alpha TV\left(D\right)+\beta ∥D{∥}_{1}+\frac{1}{2}∥\mathrm{AD}-P{∥}_{2}^{2},$$

where TV(D) represents the total variation of D, $∥D{∥}_{1}$ represents the L1‐norm of D, $∥\mathrm{AD}-P{∥}_{2}$ represents the L2‐norm of AD−P, and α and β are the relative weights of the regularization terms.

### 2.5 Dosimetry system prototype

The 3D dosimetry system is composed of a scintillator and a 3D optical measurement device. The scintillator serves as both the detection material that converts the 3D dose distribution into a 3D light distribution and the measurement phantom in which radiation deposits energy. As a detection material, the scintillator should possess a high light intensity, suitable emission spectra, a short response time, and low light absorption and scattering. As a measurement phantom, the physical density and elemental composition of the scintillator should closely resemble water (the standard measurement medium in radiation therapy) to exhibit similar radiation attenuation characteristics. Moreover, the scintillator needs to be malleable and easy to use. In addition to using scintillation gel, the measurement device also employs commercially available plastic scintillators (HND‐S2) and liquid scintillators (ULTIMA GOLD LLT).

**FIGURE 5:**
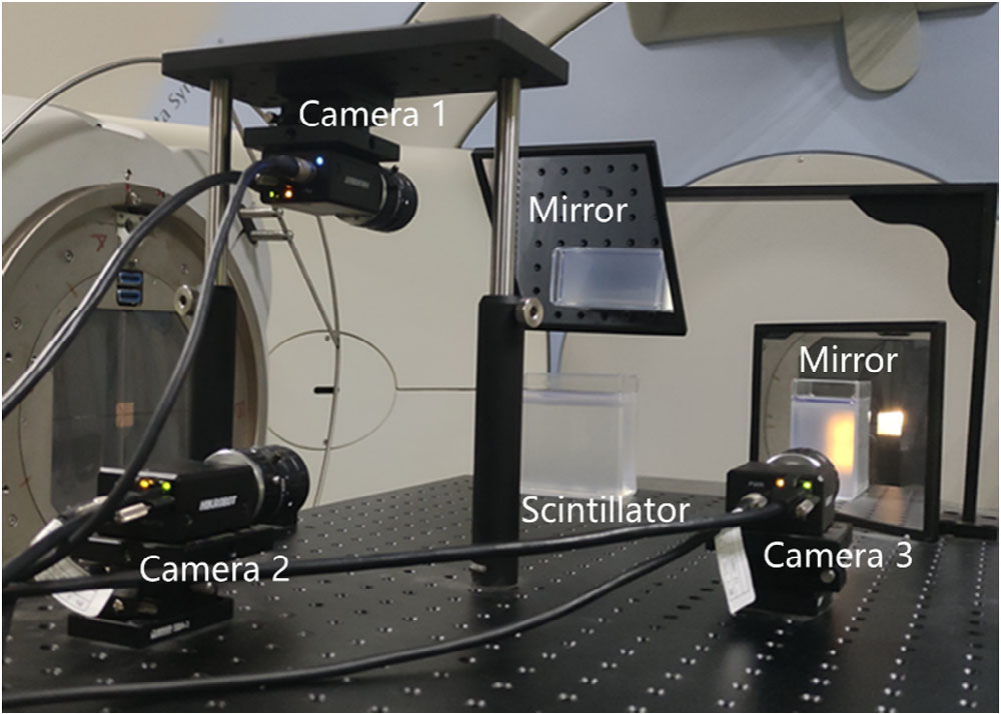
Prototype of the dosimetry system.

The prototype of the 3D dosimetry system is displayed in Figure 5.^30^ The optical measurement device includes: cameras (three orthogonal perspectives), lenses, optical brackets, reflectors, calibration boards, light shields, cables, switches, and a computer. The cameras and lenses can be replaced according to imaging accuracy and imaging distance.

**FIGURE 6:**
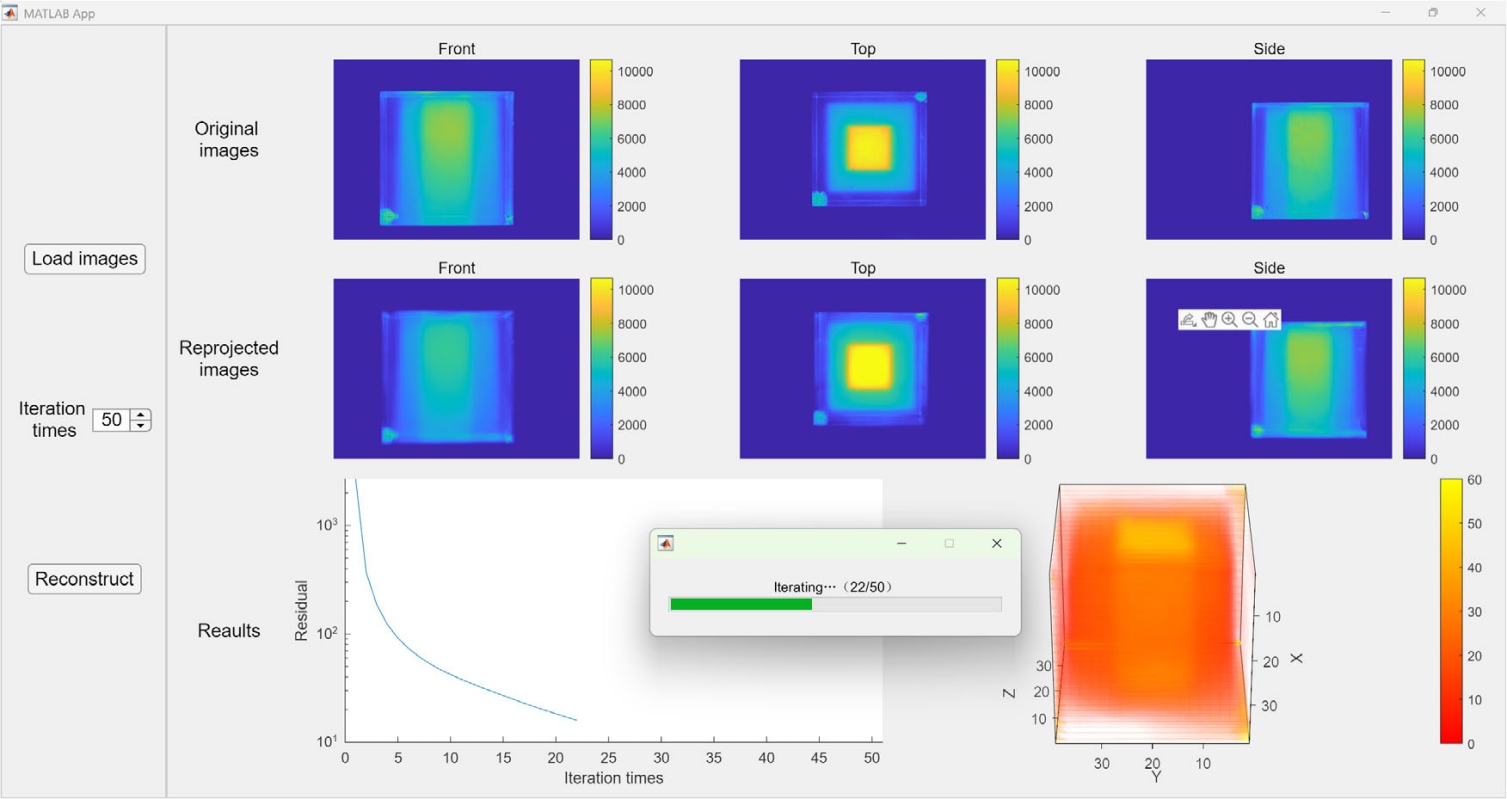
3D dose reconstruction software Gel3Ddose.

Supporting these hardware components, the dosimetry system's software comprises two parts: the image acquisition software MVS and the data processing software Gel3Ddose. In this study, the MVS software provided by Hikvision is used to synchronize the three cameras via precision time protocol (PTP). Synchronized exposure of the three cameras is achieved using soft triggers, and the time difference of the triggers can be controlled at 10 ms, ensuring synchronization with the accelerator beam output. After completing data acquisition, the data processing software Gel3Ddose is used to process and obtain the 3D dose distribution. As shown in Figure 6, Gel3Ddose is based on MATLAB. The software includes functions such as data loading, image preprocessing, equation solving, dose calibration, and results analysis. Data loading involves loading acquired images and the system response matrix. Image preprocessing primarily focuses on noise reduction. Equation solving employs the LASSO‐TV model. Dose calibration means using calibration fields to calibrate the values obtained from equation solving. The results analysis function involves comparing the measured dose with clinical reference data. In subsequent experiments, the 10×10×10 cm dose reconstruction volume was divided into 100×100×100 voxels, resulting in a reconstruction resolution of 1 mm. After data acquisition, image processing can be performed within a few minutes, enabling rapid and high‐resolution 3D dose distribution measurements.

### 2.6 Experimental setup, evaluation criteria and calibration

To test and validate the newly proposed 3D dosimetry system using scintillation gel, several experiments were conducted, such as modified segment from SBRT for intracranial tumors, and IMRT clinical plan with three‐field segmentation hypofractionation. Both SBRT and IMRT plans were performed on Elekta Synergy linear accelerator with calculation on Monaco treatment planning system (TPS).

To verify the accuracy of dose measurements, the dose measurement results of this dosimetry system were compared with those of MapCHECK2, a mainstream 2D diode array widely used in hospitals. The spatial resolution of MapCHECK2 is 7 mm. A comparison of the results was conducted using the relative error δ, expressed as shown in Equation (4):

(4) $$\delta =\frac{D_{M}-D_{R}}{D_{R}}\;\times 100\%,$$

where $D_{M}$ represents the dose obtained from the dosimetry device of this study, and $D_{R}$ represents the reference dose obtained from MapCHECK2. Typically, when the relative error δ is less than 5%, the results of the dosimetry system are considered accurate, meeting the relevant recommendations of ICRU publications.

To validate the conformity of radiotherapy plans to the requirements of reports such as AAPM TG218, it is crucial to consider both the accuracy of position and dose. In this study, the “gold standard” of the gamma passing rate^33^ was used, which evaluates the agreement between calculated and measured dose distributions. Specifically, the gamma passing rate criteria were a 5% dose threshold, a 3 mm position deviation, and a 3% dose deviation.

Calibrating the dosimetry system using a standard radiation field is essential before conducting experimental tests. In this study, prior to dose calibration, the light responses of the three cameras were normalized using a standard light source and photometer. An 8 cm × 8 cm square field uniform beam was irradiated onto the scintillator from above. The beam intensity was set to 600 MU/min, and a total of 10 irradiations were performed, with irradiation durations ranging from 1 s to 10 s in 1 s increments. Data were collected by synchronously exposing the three cameras for 10 s during each irradiation. The dose distribution obtained before calibration was denoted as $D_{0}$. For comparison with the TPS calculated results, the TPS‐calculated dose distribution for the square field uniform beam was extracted as the reference field dose $D_{\mathrm{ref}}$. For comparison with the 2D diode array, under the same irradiation conditions, MapCHECK2 was irradiated, and data were collected to obtain the reference field dose $D_{\mathrm{ref}}$. Dose calibration was performed by calculating the mean value within the central 5 cm × 5 cm region.

## 3 RESULTS

### 3.1 Validation of modified segment from the SBRT plan

Using the SBRT plan for intracranial tumor treatment, a single subfield beam with vertical incidence was applied to the scintillation gel. The measured reference field dose was acquired by irradiating MapCHECK2 with the same subfield beam. The reference field dose for the subfield beam was derived from the TPS calculations. The scintillation gel phantom of this dosimetry system can measure a volume of 10 cm × 10 cm × 10 cm. The dose measurement results were compared using three cross‐sectional planes: the coronal plane (−15.92 cm), the transverse plane (1.33 cm), and the sagittal plane (−0.40 cm). The spatial resolution of MapCHECK2 was 7.07 mm, the spatial resolution of the TPS calculations was 1 mm, and the spatial resolution of this dosimetry system was 1 mm.

Table 2 presents a comparison of dose measurements between different scintillators and MapCHECK2. When compared with the measured reference field dose obtained from MapCHECK2, the coronal plane data were considered. The threshold for dose comparison was set at 5%. The average relative error $\overset{-}{\delta }$ was calculated as the average absolute value of relative errors δ for all points above the threshold, whereas the maximum relative error $\delta_{m}$ was determined as the maximum value of relative errors δ for all points above the threshold. Because MapCHECK2 samples with a detection spacing of 0.707 cm, the dose measurement values needed to be sampled for comparison. The nearest dose points were selected for calculating relative errors. The experimental results revealed that the maximum relative error $\delta_{m}$ was less than 5%, confirming the accuracy of the results obtained from the 3D dosimetry system proposed in this study, and confirming that the dose measurement requirements of ICRU were met.

**TABLE 2:** Comparison between different scintillator dose measurements and MapCHECK2 measurements.

Material	Average relative error $\overset{-}{\delta }$/%	Maximum relative error $\delta_{m}$/%
Plastic scintillator	1.31	3.25
Liquid scintillator	1.36	3.47
Scintillation gel	1.41	3.38

Upon validating the accuracy, dose measurements of different scintillators were compared with the TPS calculated results. To assess the dose accuracy for all reconstructed dose points, 2D and 3D gamma analyses were performed. For 2D gamma analysis, the maximum reconstruction area of 10 cm × 10 cm was selected on the three cross‐sectional planes, leading to a total of 3 × 100 × 100 dose points for calculating the gamma passing rate. For 3D gamma analysis, the entire reconstruction volume of 10 cm × 10 cm × 10 cm was selected, corresponding to 100 × 100 × 100 dose points. The results are listed in Table 3. The gamma passing rates for all three planes and the whole volume exceeded 90%, meeting the requirements for clinical practice.

**TABLE 3:** Comparison of gamma passing rate (3 mm/3%) between different scintillator dose measurements and TPS calculations.

	Gamma passing rate/%			
	2D planar analysis			
Material	Coronal	Transverse	Sagittal	Volumetric 3D analysis
Plastic scintillator	98.68	97.69	94.32	98.71
Liquid scintillator	98.45	96.58	95.18	98.64
Scintillation gel	98.38	96.73	94.57	98.47

Abbreviation: TPS, treatment planning system.

**FIGURE 7:**
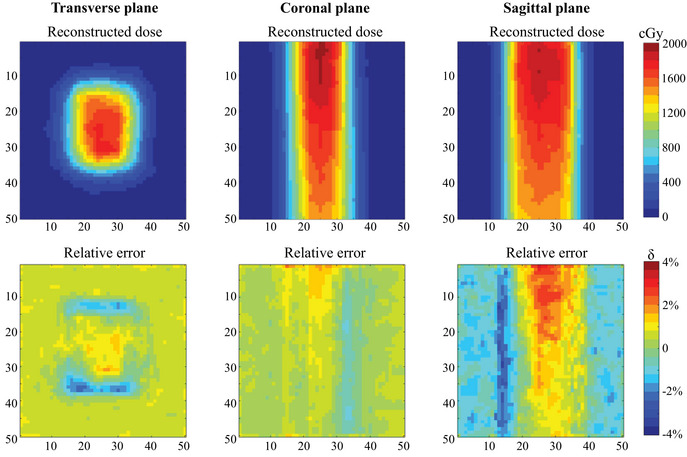
Relative error of dose data between this dosimetry system and TPS for modified segment from the SBRT plan. SBRT, stereotactic body radiotherapy; TPS, treatment planning system.

Considering the TPS calculated results had a resolution of 1 mm, and the region of interest for this radiotherapy plan was approximately 3 cm, to enhance the display effect and highlight the region of interest, the central 5 cm × 5 cm area of the three cross‐sectional planes was selected, as shown in Figure 7. A total of 50 × 50 dose points were used for the dose display and relative error calculations. The results revealed that the relative error for all measurement points was less than 5%, indicating that this radiotherapy plan met the clinical requirements for 3D dose verification. Therefore, the formulation and implementation of this radiotherapy plan were deemed appropriate.

### 3.2 Validation of IMRT clinical plan with three‐field segmentation hypofractionation

**FIGURE 8:**
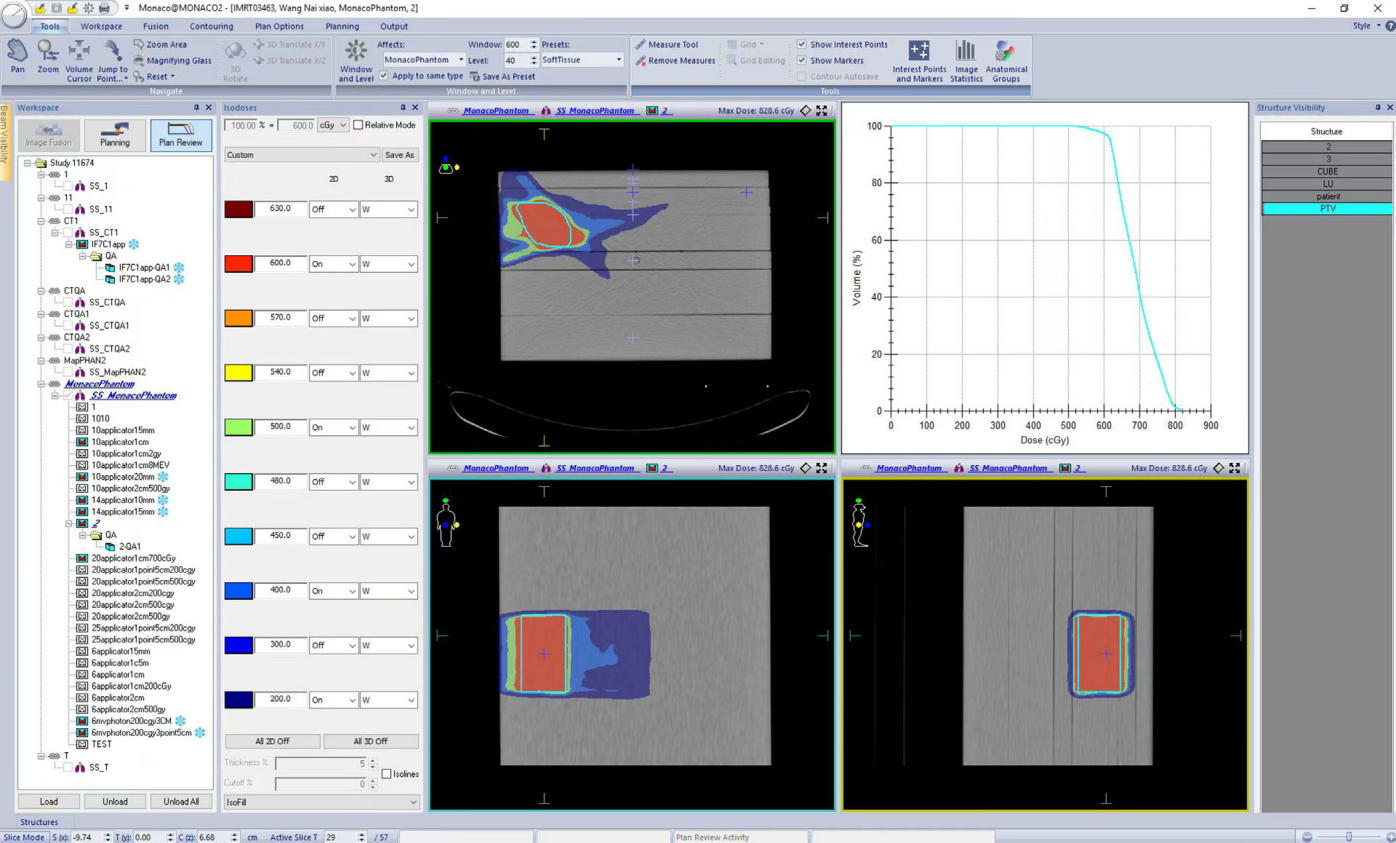
IMRT plan with three‐field segmentation hypofractionation. IMRT, intensity‐modulated radiation therapy.

**FIGURE 9:**
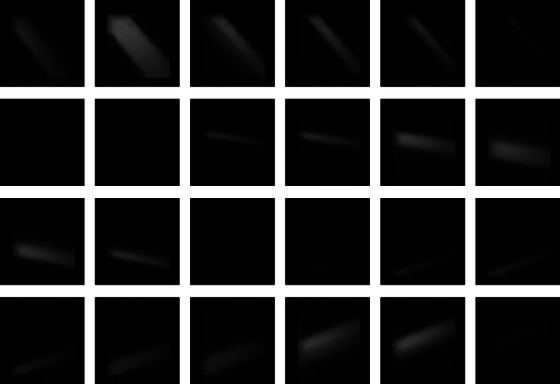
Time‐resolved image acquisition from camera 2 (front view).

The IMRT plan in this study comprised three subfields, with irradiation directions at 270° (vertical downward irradiation), 230°, and 310°. Each subfield was irradiated for approximately 1 MU, as shown in Figure 8. Time‐resolved image acquisition from camera 2 (front view) is shown in Figure 9.

**FIGURE 10:**
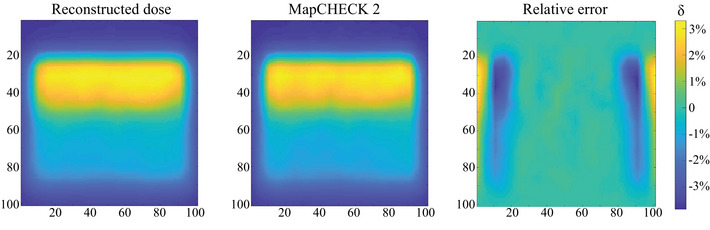
Relative error of dose data between this dosimetry system and MapCHECK2.

Following vertical irradiation of subfield 1, the reconstructed dose values of different scintillations were compared with the measured values from MapCHECK2 placed at the isocenter. The relative errors are shown in Figure 10. The error values at various points in the central region were all within 5%, verifying the accuracy of the 3D dosimetry system and complying with the requirements of ICRU measurements. Moreover, the results indicated that scintillation‐based measurement techniques can be utilized in dose verification for both a comprehensive plan and individual subfields.

**FIGURE 11:**
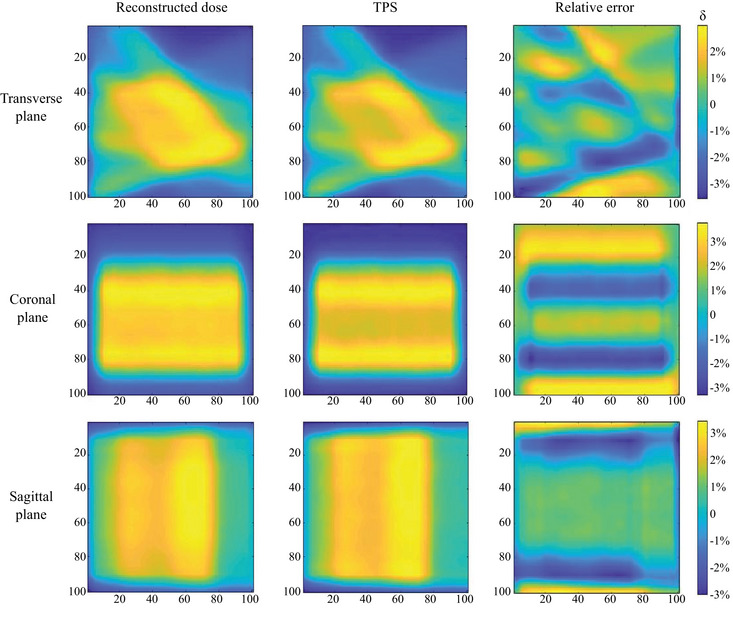
Relative error of dose data between this dosimetry system and TPS for IMRT. IMRT, intensity‐modulated radiation therapy; TPS, treatment planning system.

Based on the accuracy verification, the differences between the measurement results of this dosimetry system and the TPS calculated data were compared. The spatial resolution of the system under this plan was 2 mm. In the three cross sections, the maximum reconstruction area of 10 cm × 10 cm was selected, accounting for 3 × 50 × 50 dose points for relative error calculations. The results are illustrated in Figure 11. The relative errors for most points were within 5%, with a few points at the edge showing dose errors at around 6%. For the whole volume, the gamma passing rate was 91.14%, with even higher passing rate observed in the central region. This indicated that the IMRT plan generally met clinical requirements.

## 4 ANALYSIS AND DISCUSSION

### 4.1 Advancements of this 3D dosimetry system

The newly developed scintillation gel not only exhibited excellent scintillation luminescence and dose measurement performance but also demonstrated favorable soft tissue equivalence and moldability, serving both as a physical phantom and as a radiation detector. The established 3D dosimetry system can execute 3D dose determinations for multiple radiotherapy scenarios with a spatial resolution of 1–2 mm.

Furthermore, this dosimetry system executed dose measurement in a modified segment from the SBRT plan and IMRT clinical plan, with relative errors compared to the mainstream tool, MapCHECK2, within 5%. Moreover, although not explicitly emphasized in the measurement results, the measured 3D dose distribution was obtained as a function of time, thus achieving rapid measurements of dynamic dose distributions.

As shown in Figure 5, this work employed three cameras arranged in orthogonal perspectives and integrated the proposed LASSO‐TV dose reconstruction algorithm. It effectively addressed challenges such as limited projection directions and large‐scale sparse matrices. Furthermore, it achieved accurate 3D dose measurements with a spatial resolution of 1–2 mm, surpassing most dosimetry systems. This level of resolution is largely sufficient to meet the current demand for millimeter‐resolution dosimetry in radiotherapy. Indeed, Fourier analysis research concluded that a dose grid resolution of 2.5 mm or lower is sufficient to detect dose errors greater than 1% in IMRT.^34^ Although this conclusion was derived for 2D dose distributions, it is expected to extend to 3D measurements.

**FIGURE 12:**
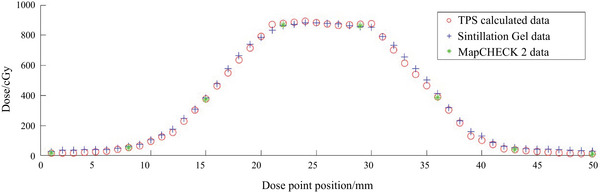
Comparison of this 3D dosimetry system, TPS, and MapCHECK2 in modified segment from the SBRT plan. SBRT, stereotactic body radiotherapy; TPS, treatment planning system.

As shown in Figure 12, during the validation of the modified segment from the SBRT plan, 50 dose points along the central axis of the coronal plane were selected for comparative analysis. The graph makes it evident that the measured data from this 3D dosimetry system exhibited minimal discrepancies compared to TPS calculations and MapCHECK2 measurements. Furthermore, differing from mainstream devices such as MapCHECK2, which have a spatial resolution of 7 mm, this dosimetry system offers a significantly superior spatial resolution of 1 mm.

Moreover, experimental testing has shown that the prototype of the 3D dosimetry system in this study exhibited no dependency on dose or dose rate. Using MATLAB, the computational time for each reconstruction on a personal computer was approximately 1 min. Nevertheless, with GPU acceleration, the reconstruction time is expected to be reduced to just a few seconds. It is also significant that the reconstruction time is proportional to the number of voxels in the scintillator volume and the number of pixels in the camera sensor. Additionally, the stability of the response over time primarily depends on variations of the scintillator optical attenuation coefficient λ over time, which tends to increase with radiation damage to the scintillator. Despite the fact that the impact of optical attenuation was minimal in the proposed method, any changes should be considered by recalibrating the detector.

### 4.2 Other factors and limitations

The estimation error of optical scattering can have a significant impact on the reconstructed dose output and should be corrected to achieve optimal accuracy. The correction proposed in this paper assumed that the optical scattering was uniformly distributed throughout the entire scintillator volume. Although this correction worked well in most cases, it may be considered too simplistic. Hence, a more detailed mathematical model of optical scattering can yield more accurate dose measurement results, providing an important direction for furthering this work. Additionally, calibration work for scintillation quenching should be considered for dose measurements of proton and heavy ion beams.

While traversing through transparent non‐vacuum media, high‐energy charged particles produce Cherenkov radiation when their velocity exceeds the speed of light in that medium. In the experiments of this study, most beams were vertically incident, and Cherenkov light in that direction was not collected by the cameras. Hence, the influence of Cherenkov radiation on the measurement results was neglected. However, in clinical practice, beams are incident from multiple angles, and Cherenkov light may affect the collection of scintillation light at specific angles. Thus, correction for Cherenkov light should be considered during dose reconstruction.

The shorter the camera acquisition time, the more data noise there is in each camera pixel, and the longer the time required for the 3D dose reconstruction of the entire treatment plan. In this study, the camera acquisition rate was one image per second, as a compromise solution to address this problem. Furthermore, in this dosimetry system, the optical panel and bracket were made of an aluminum alloy. If the radiation passes through the panel, it will undergo attenuation, introducing certain errors. This aspect will be optimized in the next stage of research.

## 5 CONCLUSION

In this work, a 3D dosimetry system for radiotherapy was proposed that comprised three components: scintillation material, optical measurements, and dose reconstruction. The prototype of 3D dosimetry system achieved high spatial resolution in the millimeter range, with time‐resolved and accurate measurements of 3D doses. A comparison with the mainstream MapCHECK2 in the field of dose verification revealed a relative measurement error within 5%, confirming the accuracy of this dosimetry system. Moreover, testing and validation in various radiotherapy scenarios, including modified segment from the SBRT plan and IMRT clinical plan, demonstrated the promising potential of this dosimetry system for dynamic treatment quality assurance.

## AUTHOR CONTRIBUTIONS

**Hua Li**: Conceptualization; methodology; writing. **Haijing Jin**: Software; formal analysis; investigation. **Liang He**: Software; formal analysis; visualization. **Xuewen Yan**: Data curation; validation. **Hui Zhang**: Resources. **Deyuan Li**: Project administration; supervision.

## CONFLICT OF INTEREST STATEMENT

The authors declare no conflicts of interest.
